# Polyurethane Microstructures for 2′-Deoxycytidinic Acid Delivery: Preparation and Preliminary Characterization

**DOI:** 10.3390/medicina60030491

**Published:** 2024-03-16

**Authors:** Roxana Maria Jeleriu, Bogdana Cavaloiu, Lidia Manuela Onofrei, Florin Borcan, Ramona Carmen Albulescu, Maria Puiu

**Affiliations:** 1 PhD School, Faculty of Medicine, Department of Microscopic Morphology, Genetics Discipline, Center of Genomic Medicine, “Victor Babes” University of Medicine and Pharmacy Timisoara, 2 E. Murgu, Sq., 300041 Timisoara, Romania; jeleriu.roxana@yahoo.com (R.M.J.); bogdana.cavaloiu@gmail.com (B.C.); lidia.onofrei@ms.ro (L.M.O.); 2 Department of Radiology, “Victor Gomoiu” Children’s Clinical Hospital, 21 Basarabia Blvd., 022102 Bucharest, Romania; 3 Department I, Advanced Instrumental Screening Center, Faculty of Pharmacy, “Victor Babes” University of Medicine and Pharmacy, 2 E. Murgu Sq., 300041 Timisoara, Romania; 4 Department XI (Pediatrics II), Faculty of Medicine, “Victor Babes” University of Medicine and Pharmacy Timisoara, 2 E. Murgu, Sq., 300041 Timisoara, Romania; albulescu.ramona@umft.ro; 5 Department II (Microscopic Morphology), Genetics Discipline, Center of Genomic Medicine, Faculty of Medicine, “Victor Babes” University of Medicine and Pharmacy Timisoara, 2 E. Murgu, Sq., 300041 Timisoara, Romania; maria_puiu@umft.ro; 6 Clinical Emergency Hospital for Children “Louis Turcanu”, Regional Center of Medical Genetics, 2 Dr. I. Nemoianu, 300011 Timisoara, Romania

**Keywords:** carrier, cells culture, MALDI-TOF, polymer, SEM, solubility, Zetasizer

## Abstract

*Background and Objectives*: Nucleotide delivery has emerged as a noteworthy research trend in recent years because of its potential utility in addressing a range of genetic defects resulting in the presence of incorrect nucleotides. The primary goals of this research were to create and to characterize polyurethane microstructures, with the aim of utilizing them for nucleotide transport. *Materials and Methods*: Two samples were prepared using an aliphatic diisocyanate in reaction with a mixture of polyethylene glycol and polycaprolactone diol, where 2′-deoxycytidinic acid was used as the active agent and glycerol 1,2-diacetate was used as an enhancer of the aqueous solubility. The solubility, pH, size distribution, and surface charge of the samples were measured, and encapsulation efficacy and release, cell proliferation, and irritation tests on mouse skin were conducted. *Results*: The results showed almost neutral acidic–basic structures with a high heterogeneity, and a medium tendency to form clusters with non-cytotoxic and non-irritative potentials. *Conclusions*: Future research could explore the efficacy of this carrier in delivering other nucleotides, as well as investigating the long-term effects and safety of these microstructures in vivo.

## 1. Introduction

The metabolism of nucleotides supports RNA synthesis and DNA replication to enable cell growth and division; however, there are multiple studies providing evidence that neurological diseases and DNA metabolism defects generate deficiencies in nucleotide salvage and synthesis [1]. Dietary nucleotide supplementation has important advantages in metabolism, immune function, healthy aging, advanced recovery under stress, and decreased risk of infection during surgery [2]. The investigations in this domain are very recent [3,4,5] and reflect the important role of this theme.

**Figure 1:**
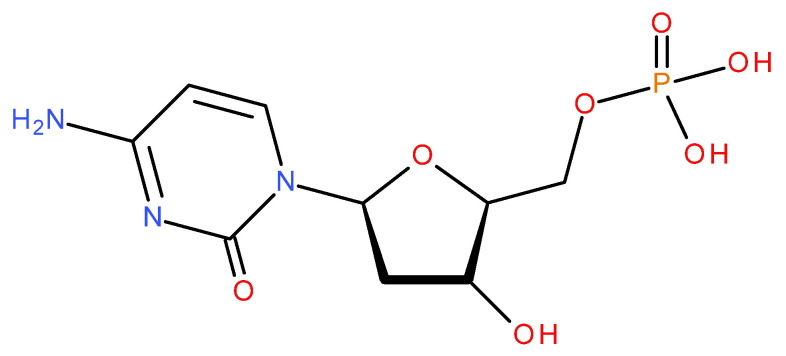
Chemical structure of 2′-Deoxycytidinic acid.

The nucleotide 2′-Deoxycytidinic acid, also known as 2’-Deoxycytidine-5’-monophosphate (Figure 1), is composed of cytidine, deoxyribose sugar, and phosphoric acid. It is formed by the reduction of cytidine monophosphate, which appears in the degradation of cytidine triphosphate and cytidine diphosphate via hydrolysis [6].

The possibility of transferring biologically active compounds through body membranes depends on the good permeation rates of the pure compound, its conjugation in various complexes, or being encapsulated inside different delivery systems. Nucleotide-based conjugates have recently been developed by Palluk et al. [7], Balintova et al. [8], and Welter and Marx [9], whereas their encapsulation has been reported by Nakayama et al. [10], Hillaireau et al. [11], Kholmurodov et al. [12], and many other research teams.

Polyurethanes (PU)—as biocompatible, pH-responsive, and biodegradable smart drug delivery carriers based on cheap raw materials—were described by Shoaib et al. [13], who presented an enhanced release rate of imatinib due to the fast degradation of polycaprolactone used as the main component of the hydroxylic phase followed by strong in vitro and in vivo antitumor effects, as suggested by MTT assay data and histopathological evaluations on BALB/c mice, respectively. The versatility of PU materials is based on the fact that they can be used in all types of administration routes; for example, PU dendrimers have been developed as drug carriers for the stable nitroxide free radical 2,2,6,6-tetramethylpiperidinyl-1-oxy [14]. The literature presents PU as a safe, compatible, and biodegradable blood-contacting material [15,16,17,18].

In our previous studies, polyurethane nano- and microstructures were developed as transmembrane delivery systems for different active agents [19,20,21,22], but all these investigations have revealed their aqueous solubility as an important disadvantage. Therefore, the main aim of this research was the design and assessment of a PU carrier that can ensure the prolonged and sustained release of a nucleotide and, more than that, the synthesized PU microstructures must have an enhanced solubility.

## 2. Materials and Methods

### 2.1. Reagents, Cells, and Animals

All the reagents used for the synthesis and degradation medium were of analytical purity and employed without previous purification: the surfactant, Tween^®^ 20 (C_18_H_34_O_6_ MW = 346.46 g/mol), was purchased from Sigma Aldrich (La Chapelle-sur-Erdre, France); isophorone diisocyanate, IPDI (C_12_H_18_N_2_O_2_ MW = 222.28 g/mol), polyethylene glycol, PEG (C_2n_H_4n+2_O_n+1_, n = 8.2–9.1 MW = 380–420 g/mol), polycaprolactone diol, PCL (C_16_H_30_O_7_ MW ~530 g/mol), 1,6-hexanediol, HD (C_6_H_14_O_2_ MW = 118.17 g/mol), tris(hydroxymethyl)amino-methane, THAM (C_4_H_11_NO_3_ MW = 121.14 g/mol), glycerol-1,2-diacetate, GDA (C_7_H_12_O MW = 176.17 g/mol), sodium chloride (NaCl MW = 58.44 g/mol), potassium chloride (KCl MW = 74.55 g/mol), magnesium chloride (MgCl_2_ MW = 95.21 g/mol), disodium hydrogen phosphate (Na_2_HPO_4_ MW = 141.96 g/mol), dipotassium hydrogen phosphate (K_2_HPO_4_ MW = 174.18 g/mol), potassium dihydrogen phosphate (KH_2_PO_4_ MW = 136.09 g/mol), sodium bicarbonate (NaHCO_3_ MW = 84.01 g/mol), 2′-Deoxycytidinic acid as sodium salt, DCSS (C_9_H_12_N_3_O_7_PNa_2_ MW = 351.16 g/mol) were acquired from Sigma Aldrich (St Louis, MO, USA), and the acetone from Honeywell Riedel-de Haen (Seelze, Germany). Double-distilled water was produced using Dest-4 distiller from J.P. Selecta (Barcelona, Spain).

A HDFa (Human Dermal Fibroblast) cell line, purchased from Invitrogen (Waltham, MA, USA), was involved to assess the cell proliferation; culture media, antibiotics, fetal bovine serum, phosphate buffer saline, MTT solution, dimethyl sulfoxide, microplates, culture flasks, and Pasteur pipettes were acquired from Thermo Fischer Scientific (Waltham, MA, USA). 

Female, 6–8-week-old Nu-Nu, Balb-c mice were purchased from Charles River (Sulzfeld, Germany). They were kept under standard conditions: temperature (25 ± 1 °C), humidity (55 ± 5%), 12 h light/dark cycle.

### 2.2. Synthesis of the Samples

A new variant of a polyurethane carrier was synthesized through spontaneous emulsification combined with the formation of macromolecular chains based on a procedure described in our previous studies [23,24]; the polyaddition process between an aqueous phase (PCL, BD, and PEG in double-distilled water, were sonicated using a homogenizer for 15 min) and an organic phase (IPDI in acetone dissolved under the same conditions) was based on the recipe described in Table 1. Tween^®^ 20 was used as an emulsifying agent, and DCSS as an active biological compound, whereas GDA was added only to the second sample. The mixtures of each sample were stirred at 350 rpm at room temperature for 3 h to complete the synthesis of polymer chains. No catalyst or initiator for chain formation was used.

After the synthesis process, the resulting suspensions were thoroughly washed and dried using a mixture of acetone and water (1.3:1, *v*/*v*) in order to eliminate any remaining unreacted raw materials and unentrapped drug. The two samples were placed in sterile Petri dishes and allowed to evaporate slowly at room temperature and normal pressure until they reached a constant mass. The process involved the gradual evaporation of the acetone and water.

### 2.3. Stability over Time

In the pharmaceutical industry, stability over time is a key parameter of newly obtained formulations; thus, different conditions such as temperature, pressure, and UV exposure are monitored to establish the quality of the products over a specified time period. In the current research, the changes in stability were assessed at three different temperatures (8, 25, and 40 °C) by comparing measured values of electrical conductivity and the position of absorption bands for the two synthesized samples; the electrical conductivity was measured with a Jenway Bench 4010 Conductivity meter (Staffordshire, UK), and a UVi Line 9400 Spectrophotometer SI Analytics (Mainz, Germany) was used to determine the wavelength of the maximum absorption. All determinations were performed in triplicate under the same conditions.

### 2.4. Evaluation of Solubility and pH

The main parameter assessed in this study was the solubility of the sample in water. Solubility was measured based on a procedure described in the literature in the OECD Guidelines for the Testing of Chemicals [25]. Briefly, at 20 ± 0.5 °C, a simple preliminary determination was used to found the appropriate amount of sample to be used in the final determination, as well as the time necessary to achieve saturation.

A Mettler Toledo FiveGo F2 portable pH meter (Schwerzenbach, Switzerland), equipped with an InLab^®^ Expert Go Sensor, was initially calibrated using three standard buffer solutions, and this was then used to assess the pH of the synthesized samples as aqueous solutions with the same concentration (0.88 mg mL^−1^) at 25 °C.

### 2.5. Encapsulation Efficacy

The percentage of DCSS successfully entrapped inside the PU microstructures was calculated using the below formula:

Encapsulation efficiency (EE%) = [(W_t_ − W_f_)/W_t_] × 100%, (1)

where W_f_ is the amount of free DCSS found in the mixture used to wash the samples, and W_t_ is the total quantity of drug added initially during preparation.

The quantity of DCSS found in the water–acetone mixture (W_f_) was calculated using the Beer–Lambert law at 271 nm; a calibration curve was constructed by plotting the absorption values as a function of drug concentration, as described by Equation (2):

y = 0.0447x, R^2^ = 0.9995, (2)

where: y = absorption value, x = drug concentration (µg mL^−1^), and R^2^ = the coefficient of determination.

### 2.6. The Release Rate

The cumulative drug release (CDR) was assessed by maintaining the PU microstructures in a simulated body fluid (SBF), which was prepared according to the T. Kokubo recipe [26] for 60 h. The procedure has been described in one of our previous articles [23]: a 1.5 mL aqueous solution of each sample (0.88 mg/mL) was added to a 30 mL SBF. Three 0.5 mL aliquots were withdrawn every 12 h and analyzed for drug content using spectrophotometry at 271 nm. After each withdrawal, 1.5 mL of fresh medium was added.

### 2.7. The Penetrability Assessment

The capacity to penetrate through different membranes influences the pharmacokinetics of the tested samples; this parameter was tested using a PVDF artificial membrane Spectra/Por^®^ from Fisher Scientific (Göteborg, Sweden). A Franz cell with a diameter of 15 mm, a diffusion area of 1.77 cm^2^, and a receptor volume of 12.0 mL was used; phosphate-buffered saline was used in the receptor chamber. The experiment was performed at 37 ± 1 °C, and every 12 h, 1.0 mL liquid from the receptor chamber was replaced with fresh buffer, and the maximum absorption at 358 nm was used to determine the concentration.

### 2.8. Matrix-Assisted Laser Desorption Ionization Time-of-Flight Mass Spectrometry (MALDI-TOF MS) Analysis

This technique represents a modern pathway for determining the size and size distribution of the polymer samples. A Bruker HCCA matrix based on a mixture of acetonitrile, water, and trifluoroacetic acid was used to suspend the PU microstructures; 1 µL of the suspension was added onto the target plate (MTP 384 ground steel, Bruker Daltonics) and the spots were allowed to dry at room temperature.

Mass spectra were obtained using UltrafleXtreme MALDI TOF/TOF instrument (Bruker Daltonics, Bremen, Germany). The analysis was performed in the positive ion reflectron mode in the mass range of 300–3000 m/z. Flex Control^®^ software (version 3.4) was used to acquire the data, and the following settings were used: laser frequency, 1000 Hz; sample rate and digitizer settings, 2.50 GS/s; accelerator voltage, 20 kW; extraction voltage, 18 kW; lens voltage, 7 kW; delayed extraction, 120 ns; and reflector voltage, 20 kW. A total of 1000 laser shots were used for each spectrum. The mass spectra were processed using the Flex Analysis^®^ software (version 3.4) (Bruker Daltonics).

### 2.9. Zetasizer Characterization

The size and surface charge of the PU microstructures were evaluated using a Zetasizer module, Cordouan Technol. (Pessac, France) with the following input parameters: temperature, 25 ± 1 °C; time intervals, 10 ± 3 μs; number of channels, 460 ± 30; laser power, 75 ± 5%; medium resolution; 3 measures/sequence; and Henry function, Smoluchowski.

### 2.10. Scanning Electron Microscopy (SEM)

Macroscopic aspects of samples were analyzed using a JSM-IT200 scanning electron microscope (Peabody, MA, USA). The samples were washed twice with acetone and then dried using compressed air; the analysis was performed at 10.0 kV with a magnification of 1500×.

### 2.11. Cells Proliferation

The biocompatibility of the synthesized PU microstructures was evaluated by measuring the cytotoxic effect on Human Dermal Fibroblasts, adult (HDFa cells); this is a skin cell line often used in research on skin diseases, drug delivery, and wound healing. The cells were seeded in a culture medium supplemented with fetal bovine serum and penicillin-streptomycin; the medium was changed at 2–3 days and proliferation was determined by the MTT technique. 1 × 10^5^ cells/mL were seeded into 96-well plates. Cells were allowed to adhere for 24 h in the incubator, and then the growth medium was changed with the test compounds (90 µL medium + 10 µL sample). The tests were performed in triplicate at 24 and 72 h after the treatment.

### 2.12. In Vivo Evaluation

Nu-Nu, Balb-c mice are the hairless representants of an inbred strain often used in biomedical research, and is particularly utilized in skin diseases and immunology. A total of 12 female mice were divided into three equal groups, labeled with the sample code (sample_1 and sample_2) and a reference (SLS—mice treated with a 2.0% sodium lauryl sulfate solution, used as a well-known skin irritant). The tested samples and SLS (approximately 50 µL once) were applied on the back skin every second day for 10 days, and the measurements were performed 30 min later. Changes in skin erythema and hydration were monitored using a Multiprobe Adapter System (MPA5) from Courage+Khazaka (Koln, Germany) equipped with Mexameter^®^ MX 18 and Corneometer^®^ CM 825 probes. The Ethical Committee for the Protection of Animals in Scientific Research at the “Victor Babes” University of Medicine and Pharmacy Timisoara granted approval for the application of the procedures and protocols (Approval number: 42/29.10.2021). Prior to the initiation of any intervention, the animals were subjected to an examination. Mice with any form of injury or dermatological condition in the dorsal region were not included in the study. To minimize potential suffering and ensure good animal welfare, measures were taken to address pain, hunger, thirst, malnutrition, abnormal cold or heat, fear, stress, injury, illness, and restrictions on the ability to behave normally/naturally. Non-invasive techniques were implemented to assess changes in skin parameters, reflecting the sentience of animals and the importance of considering their interests. Following the initial assessment, the animals were returned to their cages and were subsequently re-examined 48 h later.

### 2.13. Statistics

Statistical analysis was performed using the IBM SPSS v.27 (Armonk, NY, USA), and the Shapiro–Wilk test was used to verify the normality, while *t*-tests were used as parametric methods, and the Mann–Whitney U test as a nonparametric test was used to find significant differences. Statistical significance was set at *p* < 0.05. The charts were modeled using OriginPro 2024 software (OriginLab Corporation, Northampton, MA, USA).

## 3. Results

### 3.1. The Stability over Time

The degradation of PU biomaterials is a process that develops at various rates depending on their hydrolytic and thermal stability [27,28]; however, similar to other polymer materials, PU presents good stability over time, and the disposal of waste polymers is often a serious, long-term environmental issue. 

Modification of the absorption bands of the PU structures (abs.), and electrical conductivity (cond.) were monitored for 30 d at three different temperatures, and the percentage changes are displayed in Table 2. 

### 3.2. Solubility and pH of Samples

An important difference was found in the solubility of the synthesized products in water: sample_1 was 0.88 mg mL^−1^, while the second sample based on the addition of glycerol-1,2-diacetate presented a value equal to 6.59 mg mL^−1^. The pH values of the samples were quite similar: 6.71 ± 0.12 (sample_1), and 6.74 ± 0.15 (sample_2).

### 3.3. The Yield of Encapsulation

The encapsulation efficacy was calculated based on the amount of DCSS used in the synthesis and the amount of free DCSS found in the washed mixture used to purify each sample. Based on these results, a loading efficacy of 64.5% was found for sample_1 and 67.8% for sample_2.

### 3.4. Drug Release Profile

**Figure 2:**
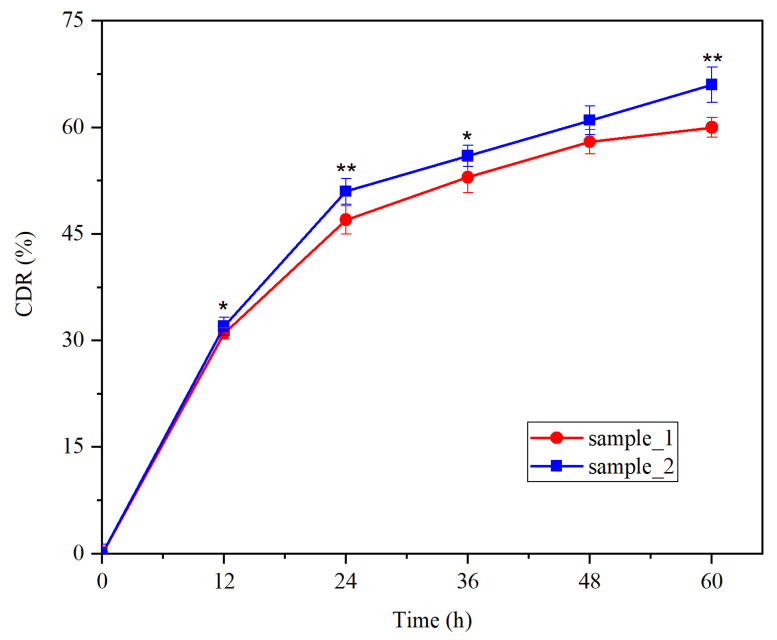
Cumulative drug release (CDR) of DCSS; * for *p* < 0.05 and ** for *p* < 0.01 sample_2 vs. sample_1.

The samples presented a biphasic release profile (Figure 2) with a fast phase up to 20 h, followed by a sustained phase. After 24 h, the release of DCSS from the synthesized PU microstructures was 47% (sample_1) and 51% (sample_2), while after 48 h, the CDR reached 58% (sample_1) and 61% (sample_2).

### 3.5. The Penetration of Membranes

**Figure 3:**
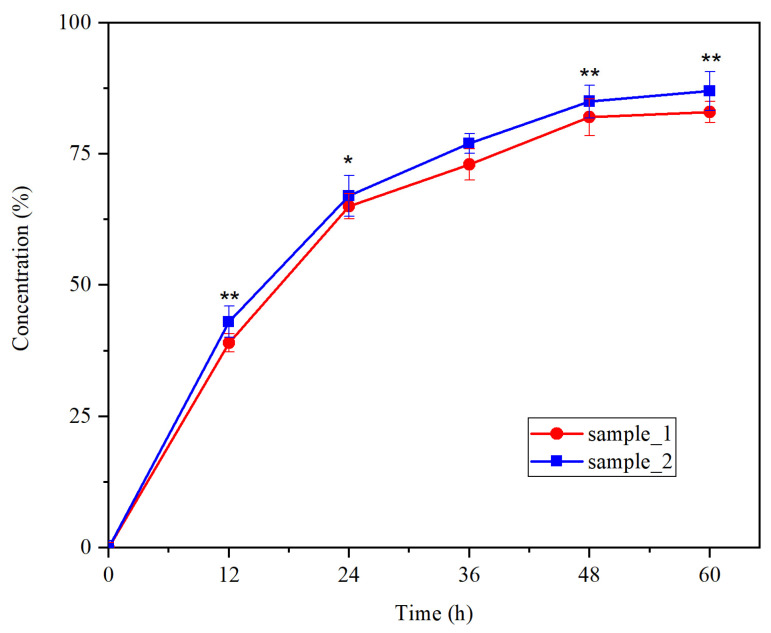
Membrane penetration rate; * for *p* < 0.05 and ** for *p* < 0.01 sample_2 vs. sample_1.

A similar permeation profile of the PU microstructures from the donor compartment through the artificial membrane into the receptor chamber of Franz cell was observed for the synthesized samples (Figure 3). It can be noted that almost 50% of the structures penetrated the artificial membrane in the first 12 h and approximately 75% passed through the membrane in the first 36 h.

### 3.6. MALDI-TOF MS Analysis

**Figure 4:**
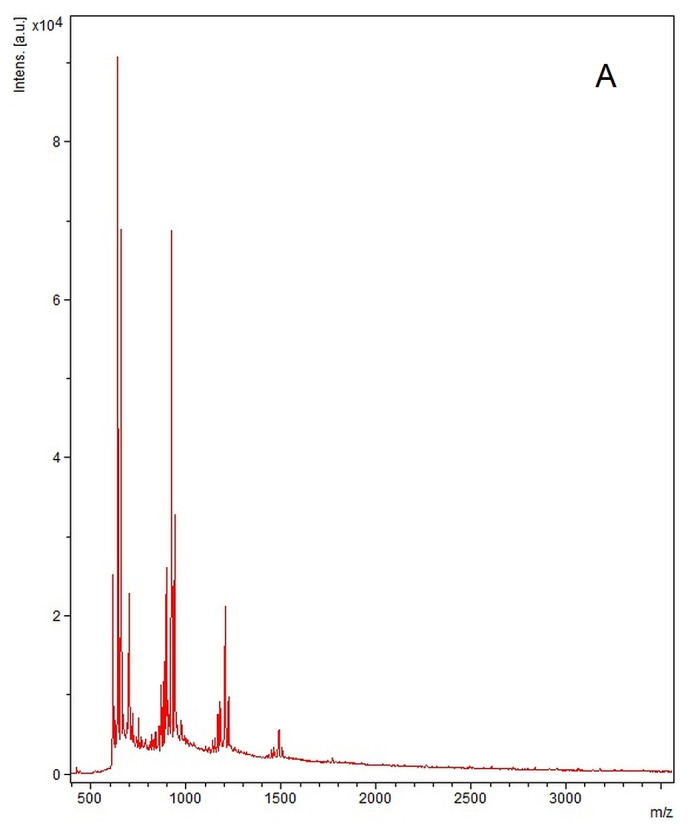

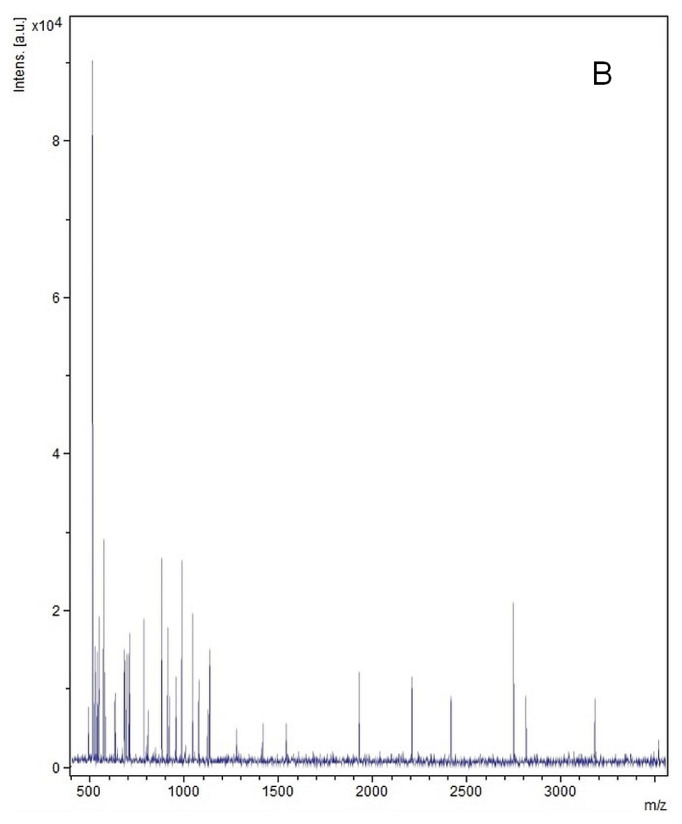
Comparative mass spectra of (**A**) sample_1 and (**B**) sample_2.

MALDI-TOF MS spectra revealed that the molecular weight of the polymer samples always presented a distribution with varied dispersity (Figure 4). The sample without GDA presented three main peaks at m/z values of 641, 925, and 1207; while the second sample was more heterogeneous and contained more populations of microstructures, with main peaks at m/z equal to 558, 640, 733, 865, 1561, 1806, 1988, and 2280. The adjacent peaks in both samples, but especially in the sample with GDA, differ in mass by one raw material unit (118.2 and 121.1 m/z for both samples due to HD and THAM, respectively 176.2 m/z due of the presence of GDA in sample_2).

### 3.7. Zetasizer Tests

The size distribution of the PU microstructures was confirmed by Zetasizer measurements. Table 3 presents data specific to multi-population samples with a medium tendency to agglomerate. 

### 3.8. SEM Investigation

**Figure 5:**
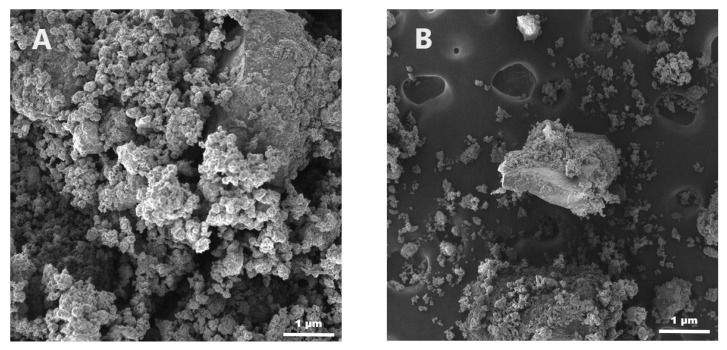
SEM images of (**A**) sample_1 and (**B**) sample_2.

The aspects of the samples (Figure 5) were studied using scanning electron microscopy at 1500× magnification. Higher homogeneity was observed in the sample without GDA.

### 3.9. Cells Culture

**Figure 6:**
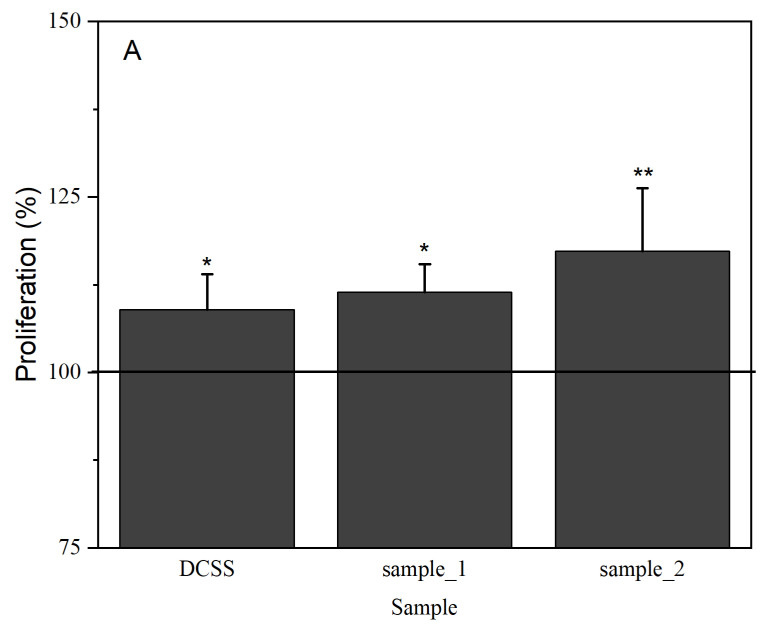

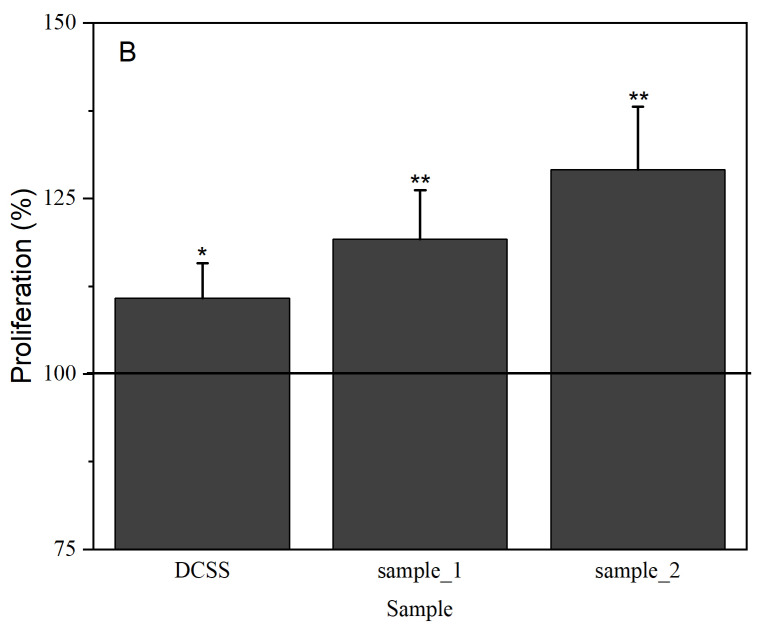
HDFa proliferation after (**A**) 24 h and (**B**) 72 h of exposure; * for *p* < 0.05 and ** for *p* < 0.01 samples vs. control.

Figure 6 presents the HDFa cell proliferation after 24 and 72 h exposure to the tested samples.

Different increases in cell viability were observed in both samples; the control was set to 100%. After 24 h, the highest value was observed for the sample containing GDA (117.3%). At 48 h post-exposure, a similar trend was observed in terms of cell viability. Thus, sample_2 exhibited the highest value compared with that of the control (129.1%).

### 3.10. Skin Irritation Evaluations

**Figure 7:**
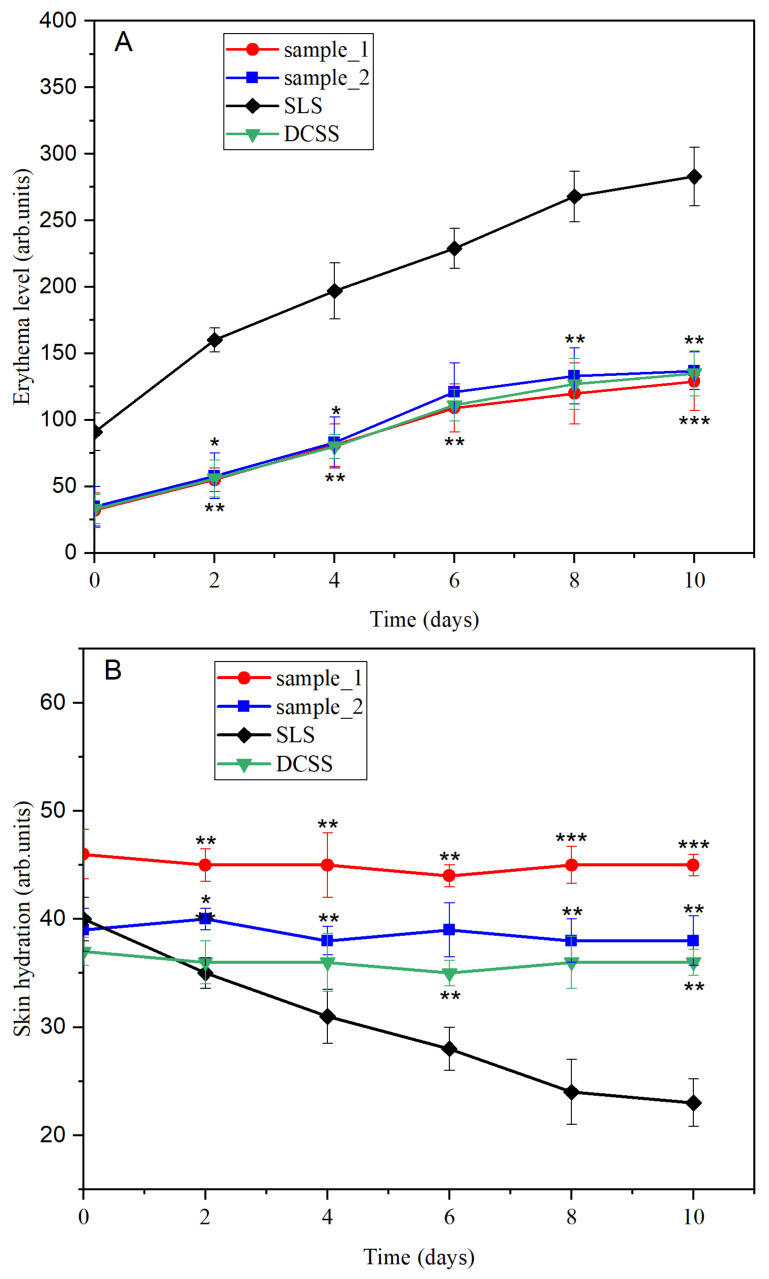
Changes in (**A**) erythema and (**B**) skin hydration during the experiment; * for *p* < 0.05, ** for *p* < 0.01, and *** for *p* < 0.001 samples vs. SLS.

Figure 7 shows the evolution of two important skin parameters that can be used as indicators of irritation effects: erythema and level of skin hydration.

## 4. Discussion

Currently, various microstructures are used as drug delivery systems owing to their notable properties, which frequently include targeted delivery, adjustable release, and high surface-to-volume ratio. Among various types of microstructures, those based on polymers have been more thoroughly investigated due to their higher encapsulation efficacy, biocompatibility, and biodegradability. The current samples are based on PU microstructures that began to be synthesized two decades ago as carriers for alpha-tocopherol by the K. Bouchemal group [29]. Despite their stability, the investigation of polymer carrier degradation is necessary to predict their half-life and release profiles. The degradation of PU biomaterials is influenced by exposure to aqueous media [30], UV [31], heat [32], and bacterial attacks [33]. As presented in Table 2, our results indicate a modification in the absorption bands between 4.0% (sample_1 at 40 °C) and 6.4% (the other sample at the same temperature). The minor changes observed can be attributed to the lack of aromatic rings and noncovalent π-interactions (orbital overlap). On the other hand, the electrical conductivity—a specific indicator of the salinity in various solutions—offers details about the incorporation of conductive segments in polymers. The electrical conductivity of PU biomaterials is approximately 2 × 10^−6^ S cm^−1^ [34], and the change in this value depends on structural modifications. The observed changes (Table 2) were between 7.3% (sample_1 at 25 °C) and 11.1% (the other sample at 8 °C).

The difference between the solubility of the two synthesized samples in water is significant; the sample without GDA presents a solubility equal to 0.88 mg mL^−1^, while in the case of the other sample was approx. 7.5x higher. The first investigation over the increase of PU particles’ solubility in water was developed by our research team four years ago and it was patented by our national authority [35]: the use of compounds with polarizable functional groups such as 1,2-propanediol, 2-acetate, or glycerol-1,2-diacetate as raw materials causes an increase of solubility. The current study revealed that this increase was proportional to the amount of auxiliary agent (GDA) added. The pH of the samples was comparable to that reported for PU carriers [19,21]. The almost neutral acidic–basic character of these microstructures is very important because it does not affect the choice of the administration route. 

Physical entrapment of different active biological compounds inside polymer delivery systems is a common approach because of its high efficiency [36]. In the current study, the highest value was registered for the second sample, with GDA (67.8%); a value that is comparable to those reported in the literature [21,23]. The DCSS release profile from PU microstructures that were maintained in SBF medium exhibited a fast phase in the first 20 h and then the cumulative percentage increased slowly from 47 to 58% (sample_1) and from 51 to 61% (sample_2) in the next 24 h. It must be noted that the degradation of PU microstructures and their release profile can be easily modified by using various polyether/polyester ratios in their synthesis [23].

The permeation rate of human membranes represents a key parameter of drug delivery systems; it has a significant impact on the pharmacokinetics of the tested formulation and depends on the molecular weight, surface charge, and transfer mechanism [37]. A low flux was found according to the classification of Ng et al. [38] on the diffusion through synthetic membranes used in a Franz cell. However, it must be mentioned that more than 75% of the PU microstructures have passed through the membranes in the first 48 h.

MALDI-TOF MS, a rapid, efficient, and accurate procedure to describe the size distribution, revealed that the synthesized samples contained multiple populations of microstructures; the first sample, without GDA, seemed to be more homogeneous, while the other sample contained more than eight populations of structures. These data were confirmed by the Zetasizer characterization: PDI above 1.0 for each sample and 3 to 6 signals in the investigation based on dynamic light scattering. Usually, PDI values are between 0 and 1, and they are equal to the ratio between the weight average molar mass (Mw) and the number average molar mass (Mn) [39]. However, this parameter, which indicates the dispersity or heterogeneity of a sample, is higher when the macromolecular chains have significant differences in their lengths. On the other hand, the literature [40] indicates that the use of PCL in the synthesis of polymer biomaterials often generates PDI > 1.

The SEM images also present data confirming the dispersity of these two samples. In Figure 5, various structures with irregular shapes can be observed; the most important difference appears in the case of the second sample based on GDA.

The proliferation and adhesion of various cells to polymer biomaterials such as polyethyleneimine, polypropyleneimine, polypyrrole, and poly(p-phenylenediamine) were reported two decades ago [41]. Zanetta et al. [42] have found that PU foams can be used as a support material for the proliferation of mesenchymal stem cells, while our previous study has revealed the same behavior [23]. In the current research, the cell viability data demonstrate the compatibility of the carrier with the cellular environment and highlight the fact that PU microstructures at the tested concentration are suitable as a delivery system for DCSS.

The evaluation of newly synthesized products on animal skin is often used because of their high sensitivity in detecting irritation potential. Currently, reproducible non-invasive techniques are used to monitor skin parameters (transepidermal water loss, erythema, hydration, pH, sebum, melanin, etc.) [43]. Kose et al. [44] reported that changes in skin parameters after the application of an irritating agent are very rapid and directly proportional to the irritation potential of the monitored agent. In this 15-day experiment, erythema evolution was characterized by an increase of approximately 113 units in the PU samples. Normally, an increase in erythema values is associated with an inflammation process, but it must be correlated with the values recorded for a known irritant; in the case of SLS application, the tested parameter has exactly doubled (226 units in the same period). An almost constant evolution of skin hydration can be observed in Figure 7 for the tested samples, whereas the application of SLS generated an important decrease in this parameter (50% lower after 15 days). Therefore, it can be concluded that the erythema and hydration values recorded for the PU microstructures indicated their low irritative potential. PU materials are also described as safe, biocompatible, and non-irritant in the literature [45].

It appears that the delivery of active molecules is a promising technology due to the unique characteristics of various drugs. However, no research can be considered completely flawless or able to account for all possible scenarios. Therefore, it is noteworthy that the sink conditions were maintained only during the initial 24 h in the evaluation of cumulative drug release. Another significant limitation of this study is the absence of a relationship between the rate of DCSS delivery and the biological effects on both cells and animals. Furthermore, it is crucial to conduct a more comprehensive investigation that incorporates histological data in order to gain a deeper understanding of the cellular and tissue-level changes that occur.

## 5. Conclusions

A significant number of drug delivery systems were developed in the last decade in order to increase the bioavailability of biologically active compounds. Since the last years of the 20th century, polyurethane carriers have been synthesized and optimized to increase the encapsulation efficacy and the solubility, and to decrease the particle size. The main objectives of this study were to grow polyurethane microstructures with enhanced solubility intended to be used as a carrier for nucleotides.

A polyaddition process between a mixture of polyethylene glycol and polycaprolactone diol on one hand and an aliphatic diisocyanate on the other, combined with an emulsification was involved in the synthesis. The results indicate that multi-population structures with an approximately neutral pH were obtained, with an enhanced solubility in water when glycerol-1,2-diacetate was used in the synthesis. Thus, this study demonstrates the potential of PU microstructures as a promising carrier for drug delivery applications, with minor changes in electrical conductivity and absorption bands over time.

Further research is needed to optimize the release profile of the drug and to investigate the long-term effects of PU microstructures on cell viability and evolution of skin parameters. Additionally, exploring the potential of PU microstructures for other drug delivery applications and evaluating their effectiveness in vivo would be a valuable future direction.

## Figures and Tables

**Table 1:** The amounts of raw materials used in the synthesis.

Sample	PCL (g)/THAM (g)	BD (mL)/PEG (g)	Tween^®^ 20 (mL)/IPDI (mL)	DCSS (g)	GDA (mL)
sample_1	3.0/1.2	1.8/3.5	1.5/6.0	0.5	0.0
sample_2	3.0/1.2	1.8/3.5	1.5/6.0	0.5	3.7

**Table 2:** The changes of absorption bands and electrical conductivity levels in 30 days.

Sample	Percentual Changes at Different Temperatures, %					
	8 ± 0.5 °C		25 ± 0.5 °C		40 ± 0.5 °C	
	abs.	cond.	abs.	cond.	abs.	cond.
sample_1	4.4	7.8	4.1	7.3	4.0	7.5
sample_2	6.3	11.1	6.2	9.7	6.4	10.8

**Table 3:** Zetasizer characterization of synthesized samples.

Sample	Size, nm (Signal Intensity)	PDI ^1^	Zeta Potential, mV
sample_1	637 ± 14 (68%) 926 ± 11 (19%) 1211 ± 17 (13%)	1.2	+28.4 ± 1.2
sample_2	556 ± 9 (62%) 640 ± 11 (8%) 735 ± 11 (9%) 866 ± 16 (6%) 1545 ± 21 (7%) 2278 ± 8 (8%)	1.4	+24.9 ± 2.3

^1^ polydispersity index.

## Data Availability

The raw data supporting the conclusions of this article will be made available by the authors on request.
